# Scent rhythms in twilight-flowering *Passiflora pohlii*: osmophore structure and terpenoid volatiles

**DOI:** 10.1093/aobpla/plag030

**Published:** 2026-06-22

**Authors:** Angie Zuleidi Mayorquin, Elder Antônio Sousa Paiva, Stefan Dötterl, Reisla Oliveira, Diogo Montes Vidal, Clemens Schlindwein

**Affiliations:** Programa de Pós-Graduação em Zoologia, Laboratório Plebeia—Ecologia de Abelhas e da Polinização, Universidade Federal de Minas Gerais, Av. Antônio Carlos, 6627, Pampulha, Belo Horizonte, MG 31270-901, Brazil; Departamento de Botânica, Instituto de Ciências Biológicas, Universidade Federal de Minas Gerais, Av. Antônio Carlos, 6627, Pampulha, Belo Horizonte, MG31270-901, Brazil; Department of Environment & Biodiversity, Plant Ecology, University of Salzburg, Hellbrunnerstr. 34, Salzburg 5020, Austria; Departamento de Genética, Ecologia e Evolução, Universidade Federal de Minas Gerais, Av. Antônio Carlos, 6627, Pampulha, Belo Horizonte, MG 31270-901, Brazil; Departamento de Química, Instituto de Ciências Exatas, Universidade Federal de Minas Gerais, Av. Antônio Carlos, 6627, Pampulha, Belo Horizonte MG 31270-901, Brazil; Departamento de Botânica, Instituto de Ciências Biológicas, Universidade Federal de Minas Gerais, Av. Antônio Carlos, 6627, Pampulha, Belo Horizonte, MG31270-901, Brazil

**Keywords:** crepuscular pollination, floral volatiles, osmophore ultrastructure, terpenoid scent emission, circadian floral rhythm, *Passiflora*

## Abstract

Some plants emit floral scents at night and are pollinated by nocturnal bees, yet their scent dynamics, composition, and emitting tissues remain poorly understood. Here, we study *Passiflora pohlii*, a passionflower whose blossoms open at dawn, are pollinated by crepuscular *Ptiloglossa* bees, and emit a strong citrus-like scent to (i) identify the chemical composition of the floral scent, (ii) quantify the rhythm of volatile emissions, and (iii) localize and characterize the ultrastructure of osmophores, to understand better their role in synchronizing pollination within a restricted temporal window. Osmophores were localized using morphochemical techniques and characterized by cellular structure. Volatile compounds were collected by dynamic headspace at early (dawn; complete and dissected flowers) and late (early morning; complete flowers) anthesis, and analysed by gas chromatography–mass spectrometry (GC–MS). Sequential ultrastructural analyses tracked subcellular changes correlated with scent emission. The outer corona filaments were the main scent source, containing osmophores and releasing volatiles. Scent emission peaked at dawn, temporally aligned with the activity of *Ptiloglossa* pollinators, before declining by 90% in late anthesis, despite unchanged floral turgor. TEM revealed organelle degradation, correlating with the decline in scent emission. The scent profile was dominated by monoterpenoids, primarily ‘citronelloids’ (geraniol and derivatives thereof). We identify a coordinated system of structural, chemical, and temporal characteristics in a species of passionflower that appear to be tailored to the sensory characteristics, body size, and crepuscular flight activities of the pollinating *Ptiloglossa* bees. These findings emphasize the importance of chemical communication in narrow plant–pollinator associations involving crepuscular bees.

## Teaser

The small white flowers of Passiflora pohlii open at dawn and are pollinated by bees active during twilight. They emit a strong citrus-like scent, rich in citronelloid monoterpenes, only from first light until sunrise, precisely when these bees forage. The fragrance is produced by osmophores on the outer corona filaments, which surround the nectar-producing center. Together, floral shape, scent, and timing appear finely adapted to attract these specialized pollinators.

## Introduction

Floral scents play a crucial role as long-distance signals in interactions between plants and a wide range of pollinator groups, particularly, but not exclusively, in environments with dense vegetation and low-light conditions (Raguso 2008, Dötterl and Vereecken 2010). Floral scents are therefore of fundamental importance for the formation of the olfactory search image of pollinators (Cordeiro et al. 2017). Through their role in multimodal synergy, which links colours, shapes, and floral rewards, they promote associative learning and flower constancy in pollinators and enhance the plant's reproductive success (Haverkamp et al. 2016, Chapman et al. 2023, Araújo et al. 2024, Jordan et al. 2024).

In close range to floral resources such as pollen or nectar, scent emission has been shown to function as a short-distance olfactory guide (Dobson et al. 1999, Howell and Alarcón 2007, Dötterl and Vereecken 2010). Identifying the site within the flower where the scent is produced thus contributes to our understanding of chemical communication between plants and pollinators (Lo et al. 2024). It helps elucidate the internal cellular processes involved in the production of volatile compounds and allows linking specific glandular structures to the genes and enzymes responsible for biosynthesis (Toh et al. 2017).

The genus *Passiflora*, with a total of 625 species, the most species-rich within the Passifloraceae family (Dhawan et al. 2004), is known for its floral diversity in shapes and colours and for being pollinated by a variety of different animal groups. Also, floral scents are variable among species (Frankie and Vinson 1977, Koschnitzke and Sazima 1997, Amela García and Hoc 1998a, 1998b, Amela García 1999, Aponte and Jáuregui 2004), and seem to be closely related to the type of pollinators (Dobson 2006). Red *Passiflora* flowers that bloom during the day and attract hummingbirds usually lack scent. In contrast, bee-pollinated and night-blooming bat flowers generally emit a strong scent (Varassin et al. 2001). The floral scent has been studied with chemical analytical approaches for ∼80 species to date, five of which were found to be odourless (Lindberg et al. 2000, Varassin et al. 2001, Montero et al. 2016, della Cuna et al. 2018).

Floral scents are frequently produced in osmophores, which are scent glands that usually present a glandular epithelium and strata of glandular cells from the subjacent parenchyma (Vogel 1963). Osmophores are commonly found in petals and produce as well as release compounds that attract (potential) pollinators (Teichert et al. 2009, Marinho et al. 2014, Tölke et al. 2018). Sensory analyses of some *Passiflora* species by dissection of flower parts, encapsulation in containers, and sensory analyses by the human nose suggest that the floral scents originate from the corona filaments (MacDougal 1994, Varassin et al. 2001).

The corona is a distinctive floral structure of Passifloraceae originating from the hypanthium and consisting of the operculum and rows of filaments (MacDougal 1994, Hemingway et al. 2011). In many cases, these filament rows are differentiated into an inner row of short, finer filaments. In contrast, the outer filaments are more robust and can vary in number across species. The outer filaments generally serve as landing platforms and attract pollinators (Krosnick et al. 2013), whereas the inner filaments have no described function.

The comparison of the ultrastructure of the corona filaments of scented *Passiflora caerulea* with those of the scentless *Passiflora suberosa* flowers revealed differences in the secretory cells of the outer corona filaments, which are presumed to produce odour in the scented species (Amela García et al. 2007). In *P. caerulea*, these cells contain smooth endoplasmic reticulum and amyloplasts, features absent in the corresponding cells of *P. suberosa*. Such structural distinctions are believed to account for the contrasting scent-producing capabilities of the two species (Amela García et al. 2007). The floral volatile compounds, however, were not analysed in that study, which prevents comparison between the organelles and the types of compounds released.

Most known *Passiflora* species exhibit diurnal anthesis, synchronized with the activity of pollinators such as bees and hummingbirds. However, a small group displays nocturnal anthesis and is pollinated by bats (e.g. *Passiflora mucronata* and *Passiflora galbana*; Varassin et al. 2001, della Cuna et al. 2018) or by sphingid moths (e.g. *Passiflora penduliflora*; Kay 2001). An even more singular strategy is observed in species with dawn flowering, such as *Passiflora pohlii* Mast. This climber exhibits small flowers that open by 04:00 a.m. and emit a pronounced citric scent (Faria and Stehmann 2010), being exclusively pollinated by crepuscular bees of the genus *Ptiloglossa*. These bees gain a competitive advantage by foraging in near-absence of competition before the flight activity of diurnal bees (Kelber et al. 2006, Wcislo and Tierney 2009, Araújo et al. 2022). Crepuscular bees exhibit visual adaptations for low-light flight (Warrant et al. 2004, Araújo et al. 2023) and usually use the strong floral scent emitted by the nocturnal/crepuscular flowers as olfactory guides (Carvalho et al. 2012, Krug et al. 2018, Araújo et al. 2024). Thus, understanding when, where, and how floral scent is released in *P. pohlii* is crucial to elucidating its interactions with crepuscular bee pollinators.

In this study, we focus on *P. pohlii*, a species of the subgenus *Decaloba*, the second-largest lineage within *Passiflora* with 230 species, including 20 representatives in Brazil (Krosnick et al. 2013). As is characteristic of this subgenus, *P. pohlii* exhibits small flowers featuring a plicate membranous operculum, two whorls of coronal filaments, and white-purple colouration. Its distribution spans from Bolivia to west-central and south-eastern Brazil, with annual flowering and short flower longevity (Faria and Stehmann 2010, Milward-De-Azevedo et al. 2012). We investigate the citric scent of *P. pohlii* flowers by addressing the following questions: (i) What is the composition of the emitted volatile compounds? (ii) When is the floral scent released? (iii) What are the sites containing floral scent-producing tissues? (iv) What is the morphology and cellular ultrastructure of the osmophores responsible for releasing the volatiles? and (v) How is the scent of *P. pohlii* composed in relation to other species of *Passiflora*? To address these questions, we used headspace sampling and gas chromatography coupled with mass spectrometry to identify volatiles at dawn and soon after sunrise, conducted olfactory tests on different parts of the flower, and performed light and transmission electron microscopy analyses of the floral tissues responsible for odour production.

## Materials and methods

### Flower structure of the study species

**Figure 1:**
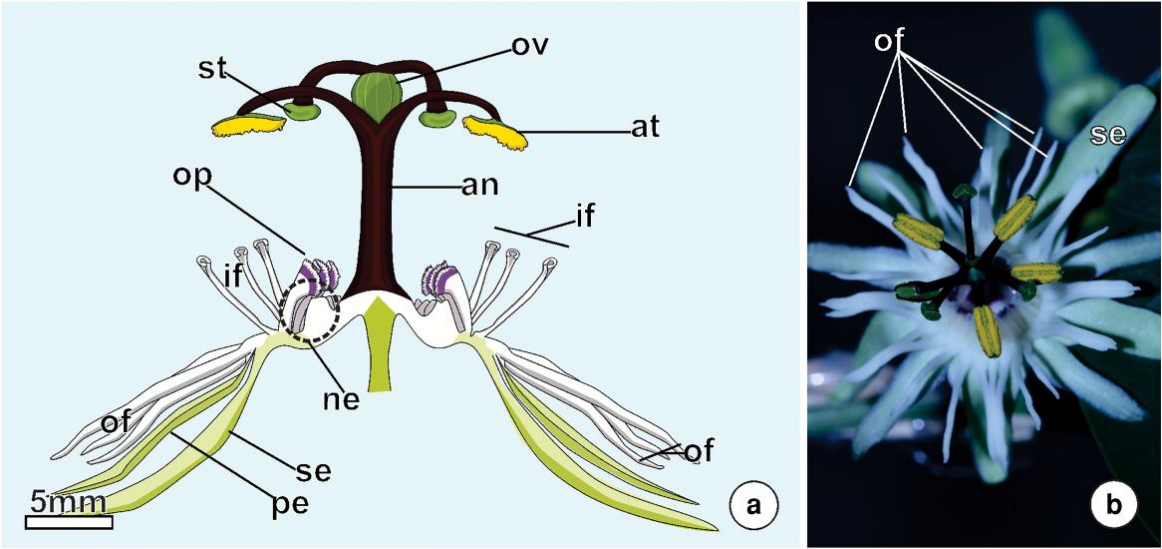
*Passiflora pohlii* flower parts. a, b) Longitudinal section of the flower and top view, respectively. Notice the different flower parts with emphasis on the long outer filaments (an—androgynophore; at—anther; if—inner filaments; ne—floral nectary; of—outer filaments; op—operculum; pe—petal; se—sepal; st—stigma).

The flowers of *P. pohlii* range from 2.5 to 4.5 cm in diameter. The corona consists of the operculum, inner filaments, which are short white filaments with capitate tips parallel to the androgynophore, and outer filaments, also white but larger than the inner filaments and perpendicular to the androgynophore (Fig. 1a and b) (Faria and Stehmann 2010).

### Floral volatiles sampling and chemical characterization

To determine the floral volatiles and quantify the intensity of floral scent emission during anthesis, we performed dynamic headspace sampling on flowers from four *P. pohlii* individuals at early anthesis (5:30–7:30 a.m., one flower per individual) and on three additional flowers at late anthesis (8:00–10:00 a.m.). *Passiflora pohlii* leaves were used as a control, and volatiles emitted by them were excluded from our analysis.

The headspace analysis was conducted by placing each sample into an odourless polyester oven bag (40 cm in length, Wyda^®^), and sampling using a vacuum pump (G12/01 EB; Rietschle Thomas, Puchheim, Germany) with an airflow rate set at 200 ml/min, regulated by a flow meter. A glass trap (30 mm in length and 3 mm in internal diameter) filled with a filter comprising glass fibre and HayeSep Porous Polymer Adsorbent (10 mg, particle size 100–120 mesh) was used to capture the volatile compounds from the samples.

The capillary, housing the filter with the volatiles, was eluted with 200 µl of solvent (acetone or hexane, HPLC grade, ≥99.9%, Sigma-Aldrich). The samples were stored at −4°C until analysed by GC–MS (Shimadzu GC2010 gas chromatograph coupled to a Shimadzu QP2010 Plus mass spectrometer (EI) using a Restek RTX-5 capillary column, 30 m × 0.25 mm × 0.25 µm). The oven temperature was held at 50°C for 1 min, increased at 7°C/min to 270°C, and held for 10 min.

Data were processed using GCMSsolution workstation software (version 4.20). Chemical structures were proposed based on mass spectral interpretation, comparison with reference spectra and retention index libraries (FFNSC and NIST), and database searches (NIST Chemistry WebBook and SciFinder). For quantification, β-pinene (100 ppm) was used as an external reference standard. Linear retention indices (RI) were determined by co-injecting 1 μl of a stock solution of straight-chain hydrocarbons (C_10_–C_26_) with 1 μl of the target compound or natural extract. The oven temperature was programmed from 50 to 270°C at 3°C/min, with a final hold of 10 min, and linear RI were calculated according to the method of van Den Dool and Kratz (1963).

To determine whether there were differences in the quantitative analyses (absolute amount of the substances) of the odour emitted between the early and late phases of anthesis, a PERMANOVA analysis was conducted in R v4.5.0 (R Core Team 2025) using the *vegan* package and the *adonis2* function (999 permutations) (Anderson 2017). Additionally, an independent samples *t*-test was performed (PRUEBA.T function, two-tailed, unequal variances) to compare the total amount of floral volatiles between the two stages (*P* < .05).

### Sensory analysis

To identify the location of the odour-emitting parts of the flower, we dissected five flower buds immediately before anthesis, into their constituent parts: sepals, petals, inner filaments, outer filaments, calyx tube (operculum and limen), and androgynophore (Fig. 1a and b). Each part was placed in an odourless polyester oven bag (40 cm, Wyda^®^) to accumulate the emitted odour for 2 hours, between 4:00 a.m. and 6:00 a.m., the period of strongest scent emission by the flowers. Thereafter, the numbered bags were subjected to five blind odour perception tests by five different human volunteers. Each individual smelled the dissected parts of a distinct flower to identify the part with the strongest odour (see Paiva et al. 2019).

### Scent analysis from outer filaments and residual floral tissues

To determine whether the outer filaments were the primary source of odour, as suggested by the previous sensory test, volatile compounds were collected separately from dissected outer filaments and residual floral tissues (flowers minus outer filaments) during early anthesis (*n* = 3 in total). For details of scent sampling and analysis, see ‘Headspace analysis of floral volatiles’ above.

### Morphology and histochemical analyses (light microscopy)

We collected flowers from three cultivated individuals of *P. pohlii* on the campus of the Federal University of Minas Gerais (UFMG, 19°52′5.628″ S 43°57′59.904″ W) and at the Museum of Natural History and Botanical Garden of UFMG (MHNJB, 19°53′31.632″ S 43°54′47.984″ W). From the three individuals, we selected three flowers per individual across the following flowering stages: (i) preanthesis, ∼5 hours before flower opening (10:00 p.m.), (ii) early anthesis: ∼2 hours after flower opening, when strong odour is perceived (5:30 a.m.), and (iii) late anthesis: ∼5 hours after flower opening when floral odour was weak (8:00 a.m.).

The flower buds and flowers were cut to obtain samples for transverse and longitudinal sections. Samples were fixed in Karnovsky fixative (Karnovsky 1965) under a vacuum of −400 mmHg, then left in this fixative for 24 hours. Subsequently, the samples were dehydrated through a graded ethanol series and embedded in 2-hydroxyethyl methacrylate historesin (Leica Microsystems Inc., Heidelberg, Germany) in a freezer at −18°C for 48 hours before polymerization, as described by Paiva et al. (2011).

Transverse and longitudinal sections (5 μm thick) were obtained with a rotary microtome (Hyrax M40, Carl Zeiss Mikroskopie, Jena, Germany), stained with 0.05% toluidine blue pH 6.8 (O’Brien et al. 1964), placed on slides, and mounted in synthetic resin Entellan (Merck, Darmstadt, Germany). To detect starch and lipids, unstained sections were subjected to histochemical tests using Lugol (Johansen 1940) and Sudan Red 7B (Brundrett et al. 1991), respectively. All sections were analysed and documented using an image capturing system (TV0.5XC-3, Olympus Scientific Solutions, Waltham, USA) coupled to a light microscope (CX41RF, Olympus Scientific Solutions, Waltham, USA).

### Ultrastructural analysis (TEM)

We conducted ultrastructural analysis of the outer corona filaments at the same stages sampled for morphological and histochemical analyses (preanthesis, early anthesis, and late anthesis). Filament segments were cut into pieces up to 1 mm long. The samples were fixed in Karnovsky fixative (pH 7.2, 0.1 M phosphate buffer) for 24 hours. The excess air was removed using a slight vacuum to improve fixative penetration. Following this, the samples underwent fixation in osmium tetroxide (1% in 0.1 M phosphate buffer, pH 7.2). Before embedding in epoxy resin, they were contrasted with a 2% aqueous solution of uranyl acetate and dehydrated in an increasing acetone series. The 50 nm ultrathin sections were contrasted with lead citrate (3% w/v, 5 min) and examined with a Tecnai-G12 Spirit Transmission Electron Microscope (TEM) (Philips/FEI Company, Eindhoven, The Netherlands) at 80 kV.

### Surface structure analysis (SEM)

Filaments were initially fixed in Karnovsky’s fixative (Karnovsky 1965) before processing for scanning electron microscopy. Preparation involved dehydration in an ascending ethanol series, followed by CO_2_ critical-point drying (Robards 1978) performed with a CPD030 apparatus (Bal-Tec/Leica, Balzers Union, Liechtenstein). Dried samples were coated with ∼10 nm of a palladium–gold alloy using an SCD030 sputter coater (Bal-Tec, Balzers, Liechtenstein), and subsequently the surfaces of the inner and outer filaments were analysed using a Quanta 200 scanning electron microscope (Philips/FEI Company, Eindhoven, the Netherlands).

### Comparative analysis of floral volatiles in *Passiflora*

To contextualize the floral fragrance profile of *P. pohlii* within the genus *Passiflora*, a comparative analysis of floral compounds was conducted through a bibliographic survey in databases, using the following search strategies: Web of Science (*Passiflora*) AND (flower OR floral) AND (‘volatile compound’ OR ‘floral scent’) and Google Scholar (‘volatile compounds *Passiflora* flowers’, ‘floral scent *Passiflora*’). In total, 92 fragrance profiles from 79 species were used, compiled from seven studies (Lindberg et al. 2000: 8 species; Varassin et al. 2001: 3 species; Cutri 2013: 3 species; Shen 2013: 1 species; Montero et al. 2016: 5 species; doctoral thesis, Clifford 2017: 70 species; della Cuna et al. 2018: 1 species; see Supplementary Table S1). The selected studies presented significant methodological variations. Collection methods included dynamic headspace, static headspace, and direct extraction. Headspace collection times varied from 1 to 24 hours across studies. The adsorbents used in dynamic headspace differed among the studies (HayeSep in the present study; Tenax-TA together with Carbotrap and Porapak Q in the others), as did the solvents and the chromatographic and identification procedures. Furthermore, profiles of the same species may correspond to different populations, with possible compositional variations. Species with more than one profile were maintained as independent entities. Although variation in collection times may introduce differences in scent concentration and composition, we chose to retain all studies to ensure a representative and robust number of species in the comparative analyses. Finally, to reduce the impact of methodological heterogeneity, we opted to use relative percentages of compound classes in the analyses, which allowed us to homogenize comparisons across studies with different analytical sensitivities. All compound tables were standardized in a spreadsheet, and quantitative data were converted to percentages. To reduce data dimensionality and minimize interference from sporadic components, compounds with relative abundance below 2% in all samples were excluded from the analyses, resulting in a final set of 318 compounds for statistical processing.

The relative abundances of all compounds belonging to the same class within each species were summed. Thus, a table was obtained containing the compound classes, the species, and the percentage corresponding to each class in each species.

Analyses were performed in R v4.5.0 (R Core Team 2025). Relative abundances of compound classes were CLR-transformed using the compositions package (van den Boogaart et al. 2024) to correct for compositional bias (Aitchison 1982). Null values were replaced with 0.001 via dplyr (Wickham et al. 2023) before transformation.

Chemical dissimilarity among flowers was calculated using Euclidean distance transformed data. Hierarchical clustering (Ward.D2) was performed with the hclust function and dendrogram was visualized using factoextra (Kassambara and Mundt 2020). Cluster robustness was assessed by bootstrap analysis (1000 replicates) with pvclust (Suzuki et al. 2019) after data transposition. Branches with AU values ≥95% were considered well-supported.

To demonstrate the percentage of each compound type in the different clades of the dendrogram, the species were grouped by branch. Then, the average of the compound classes for each branch was calculated, and a pie chart was created to represent the percentages of the most abundant classes visually on the dendrogram.

## Results

### Floral volatiles at early anthesis of complete flowers

The floral scent of *P. pohlii* contained a total of nine terpenoids. Thereof, five compounds contributed together to almost 93% of the total scent: β-citronellol (37%), geranial (36%), geranyl acetate (9%), geraniol (6%), and neral (5%) (Table 1).

**Table 1:** Absolute value (ng/flower/hour, mean ± SD) and relative value (percentage, mean ± SD) of compounds found in floral scent headspace samples of *Passiflora pohlii* flowers during early anthesis stage (0530–0730 h), and late anthesis stage (0800–1000 h).

Compounds	RI	Early anthesis (*n* = 4)		Late anthesis (*n* = 3)	
		Absolute value (ng/flower/hour)	Relative value (%)	Absolute value (ng/flower/hour)	Relative value (%)
**Terpenoid**					
Citronellal	1156	303 ± 196	1 ± 0.2	8 ± 13	0.2 ± 0.3
β-Citronellol	1233	10 040 ± 5025	**37** ± **9.5**	536 ± 498	21 ± 26.1
Neral	1247	1574 ± 1061	**5** ± **0.7**	265 ± 359	9 ± 5.2
Geraniol	1261	1687 ± 1585	**6** ± **2.8**	148 ± 189	4 ± 3
Geranial	1279	10 543 ± 6007	**36** ± **4.2**	1117 ± 1024	55 ± 23.1
Methyl geranate	1333	70 ± 77	0.3 ± 0.5	10 ± 16	0.5 ± 0.8
Citronellyl acetate	1357	778 ± 438	3 ± 0.8	28 ± 29	0.9 ± 0.6
Geranic acid	1372	828 ± 743	3 ± 2.3	54 ± 66	2 ± 1
Geranyl acetate	1387	2708 ± 2268	**9** ± **6.3**	210 ± 315	7 ± 5.4

Values >5% are printed in bold. RI, retention index.

### Schedule of odour emission

**Figure 2:**
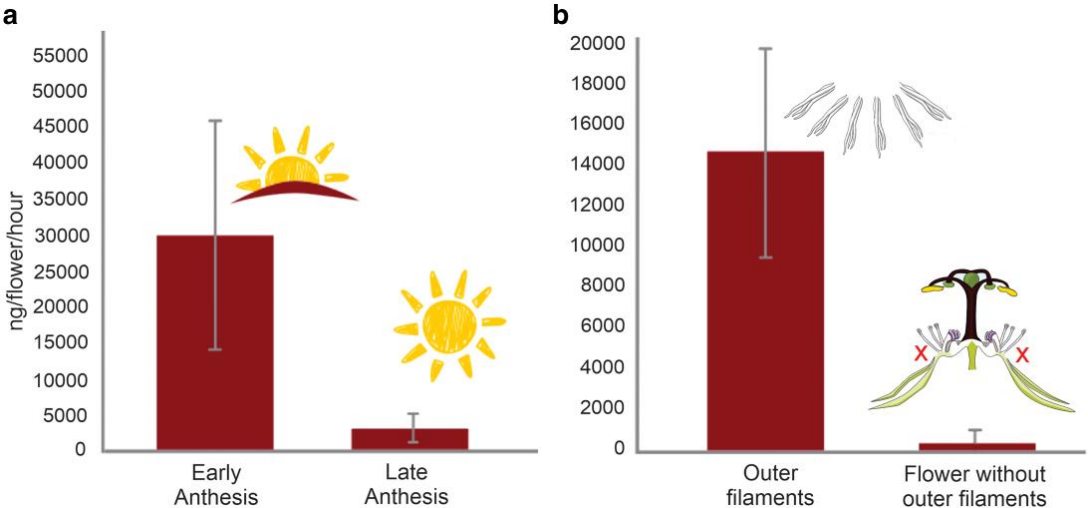
Scent emission quantity by time and in outer filaments versus the rest of the flower. a) Total amount of compounds (ng/flower/hour) produced in the early (5:30 –7:30 a.m.) and late (8:00 –10:00 a.m.) stages of anthesis of *Passiflora pohlii*. b) Total amount of compounds emitted (ng/flower/hour) by the outer filaments compared to those emitted by the whole flower, with the outer filaments removed, demonstrating that the outer filaments are the primary source of odour in *Passiflora pohlii* flowers.

The flowers of *P. pohlii* began to open around 03:30 a.m. and were fully open by 04:00 a.m.; however, they did not emit any noticeable scent for the human nose. The scent emission started around 05:15 a.m. Our findings indicate that the chemical composition of the floral scent differed significantly (PERMANOVA, *F* = 6.5907, *P* = .041) between early anthesis (05:30–07:30 a.m.; twilight hours) and late anthesis (08:00–10:00 a.m.; daylight) despite the presence of the same compounds in both stages (Table 1). This difference in composition is likely due to proportional changes in the relative abundances of individual compounds. In fact, an independent samples *t*-test revealed that the total amount of floral volatiles was significantly lower in late anthesis (*P* = .044), corresponding to an approximate 89.96% reduction (±54.12%) following dawn (Fig. 2a), even though the flower remained fully turgid. Flower wilting started around noon, with the petals and sepals bending upward covering stamens and gynoecium.

### Sensory analysis

The sensory analysis of all five persons indicated that the outer filaments of the corona emitted the distinctive citric aroma that is typical of the entire flower. This odour contrasted with all other flower parts, which exhibited either a minimal scent or whose scent was entirely undetectable to human olfaction.

### Floral scent emission in outer filaments and in flowers with these filaments removed

Headspace sampling and chemical analyses of dissected outer coronal filaments in *P. pohlii* (Table 2) revealed terpenoid profiles similar to whole-flower volatile emissions (Table 1). Experimental removal of these filaments reduced total scent emission to residual levels; only four volatile compounds (collectively representing 2% of the outer filaments’ emission) were detected after removal of the outer filaments in one of three replicate samples (Fig. 2b), with no volatiles were detected in the other two biological replicates.

**Table 2:** Location of floral scent emission in *Passiflora pohlii*.

Compounds	RI	Outer filaments (*n* = 3)		Flower without outer filaments (*n* = 3)	
		Absolute value (ng/flower/hour)	Relative value (%)	Absolute value (ng/flower/hour)	Relative value (%)
**Terpenoid**					
γ-Isogeraniol	1146	42 ± 17	0.3 ± 0	0 ± 0	0 ± 0
Citronellal	1156	80 ± 25	1 ± 0.1	0 ± 0	0 ± 0
β-Citronellol	1233	5243 ± 1936	36 ± 2.7	0 ± 0	0 ± 0
Neral	1247	609 ± 303	4 ± 0.9	14 ± 19	1 ± 2.4
Geraniol	1261	644 ± 256	4 ± 1.9	44 ± 62	4 ± 7.8
Geranial	1279	6081 ± 1769	43 ± 3.8	269 ± 381	27 ± 47.6
Citronellyl acetate	1357	1000 ± 580	6 ± 2.6	0 ± 0	0 ± 0
Neryl acetate	1365	189 ± 157	1 ± 0.8	0 ± 0	0 ± 0
Geranyl acetate	1387	500 ± 160	4 ± 0.4	0 ± 0	0 ± 0

Quantification of volatiles emitted during the early anthesis stage from the outer filaments of the corona and from flowers where the outer filaments were experimentally removed.

Absolute amount of different volatile compounds emitted (ng/flower/hour, mean ± SD). RI, retention index.

### Structure and histochemical analyses

**Figure 3:**
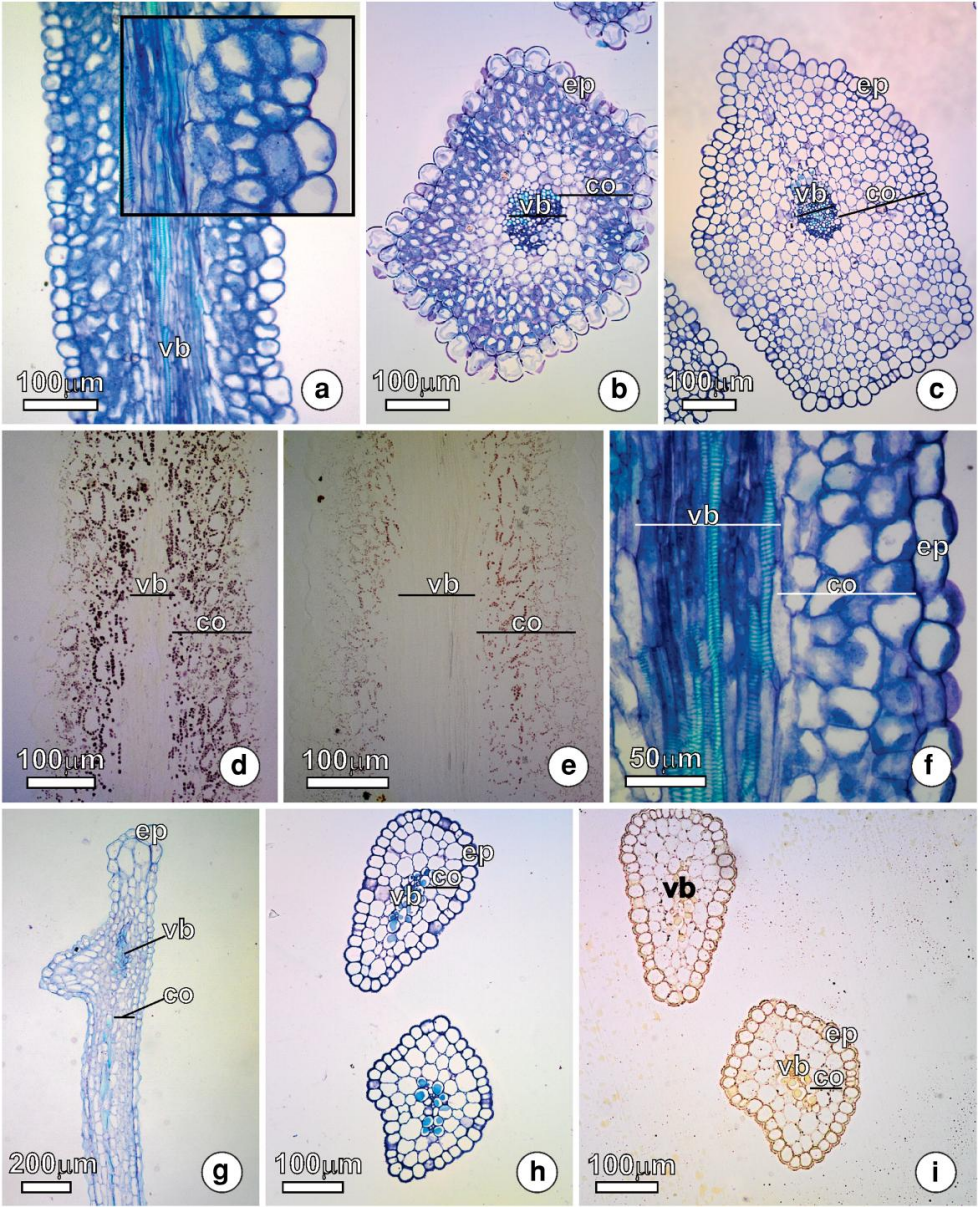
Structure of the filaments of the corona of *Passiflora pohlii.* a–f) Outer filaments. a) Longitudinal section in the preanthesis stage; notice the dense cytoplasm of the subjacent parenchyma cells (insert) and the central vascular bundle with xylem and phloem. b) Transverse section of the distal portion in preanthesis showing a central vascular bundle, and the outer cortex with dense secretory cells. c) Transverse section of the basal portion highlighting vacuolated cells through the cortex. d, e) Histochemical analysis with Lugol to detect starch (dark spots) in a longitudinal section during early anthesis. d) Filament tip showing a high density of starch grains. e) Filament base with lower starch content. f) Longitudinal section in late anthesis; note that in the cortex, the parenchyma cells show less dense cytoplasm than in preanthesis. g–i) Inner filaments at preanthesis. g) Longitudinal section in the preanthesis stage; note the overall structure similar to that of the outer filament; in the cortex, however, there are no cells with dense cytoplasm, and the large vacuole deserves highlighting. h) Transverse section of the distal portion showing a tiny central vascular bundle, and the outer cortex with vacuolated parenchyma cells. i) Transverse section of the distal portion as in h), subjected to Lugol test; the starch, restricted to minute grains, is practically imperceptible (co—cortex; ep—epidermis; vb—vascular bundle).

The outer filaments of *P. pohlii* exhibited an oval cross-section and contained a prominent central vascular bundle that extended longitudinally along the entire filament length. Notably, this vascular tissue showed no lateral branching or projections into the surrounding cortical parenchyma (Fig. 3a–c). Along the filament, in the acropetal direction, we observed an increase in potentially secretory cells in the cortex. In the basal portion, cells showed highly vacuolated and practically devoid of reserves (Fig. 3c); in contrast, in the apical portion, the cortex is entirely formed by secretory parenchyma cells (Fig. 3a).

During the early anthesis phase, the Lugol test, used to detect starch, showed a positive result, evidencing a large quantity of starch throughout the cortex of the apical portion of the outer filament (Fig. 3d), similar to that observed in preanthesis. However, in the basal portion of the filament, the presence of starch was notably lower, in addition to being restricted to the most external cortical portions (Fig. 3e). Another interesting aspect regarding starch reserves is that, in the late anthesis phase, the Lugol test yielded a negative result, indicating the absence of starch in the filaments at this stage. In addition to a reduction in starch reserves, in late anthesis, the cortical parenchyma cells show less dense cytoplasm than in the previous phases (Fig. 3f). Across all observed phases, the epidermal cells of the outer filaments maintained a consistent structure with a notably larger central vacuole than the subjacent parenchyma. Moreover, both the epidermal and subjacent parenchyma cells displayed well-developed nuclei.

The inner filaments structure overall resembles that of the outer filament, including the vascular bundle that extends to near the apex of the filament (Fig. 3g). However, in addition to the smaller diameter of these filaments, the epidermal cells are less voluminous and, above all, the parenchyma cells of the cortex are highly vacuolated and practically devoid of starch (Fig. 3h and i).

### Ultrastructural analysis of the outer filaments

#### Preanthesis

**Figure 4:**
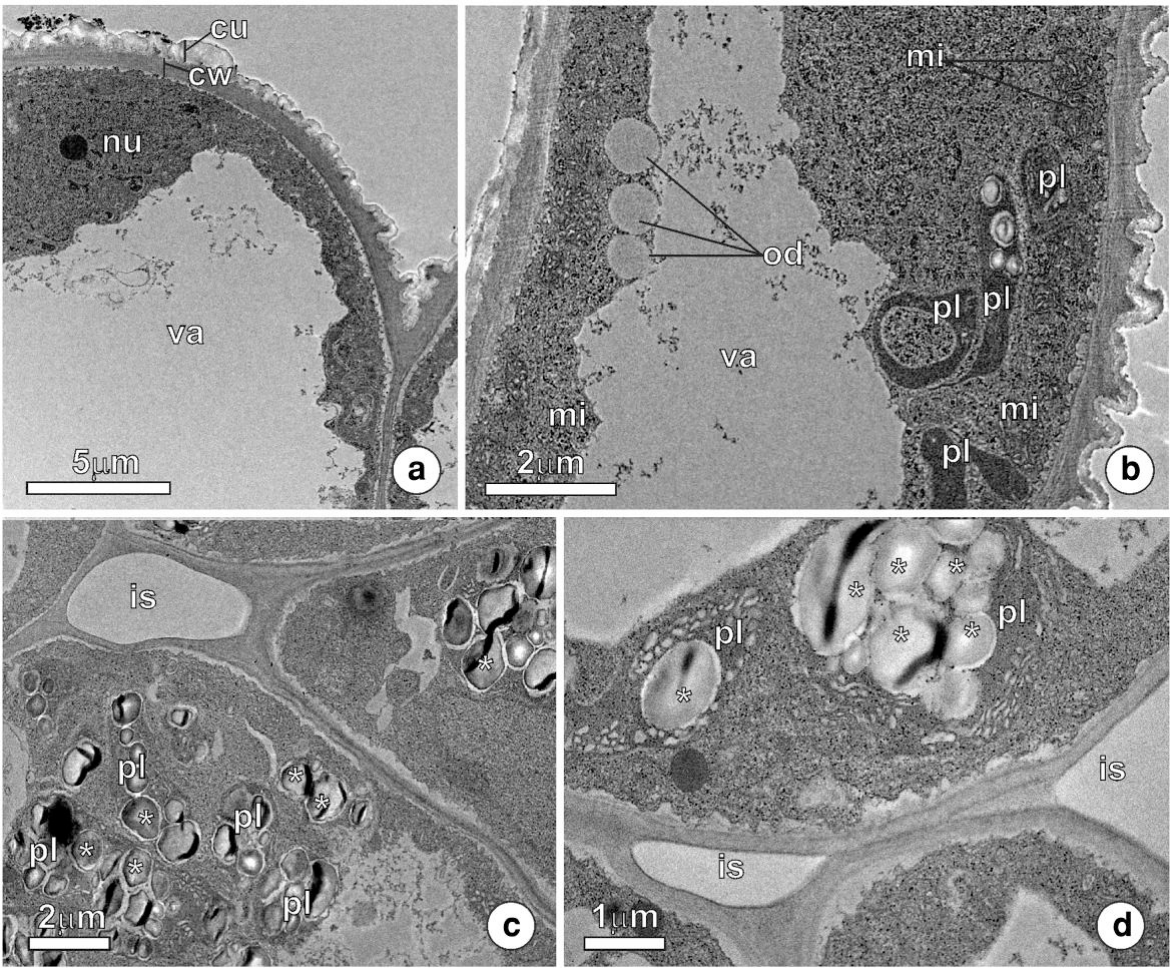
Ultrastructure of the distal portion of the outer filament of *Passiflora pohlii* in the preanthesis stage. a, b) Epidermal cell with a wavy cuticle firmly attached to the cell wall and a large central vacuole; in b), the presence of oil droplets can be seen, and mitochondria and plastids stand out. c) Secretory parenchyma cells with numerous plastids, filled with starch grains (*). d) Detail of the cytoplasmic portion of a secretory cell with large plastids filled with starch grains without signs of hydrolysis (cu—cuticle; cw—cell wall; is—intercellular space; mi—mitochondria; nu—nucleus, od—oil droplet; pl—plastid; va—vacuole).

At this stage, ultrastructural analysis of the outer filaments revealed epidermal cells with a wavy cuticle firmly attached to the cell wall. They exhibited a larger central vacuole than those of the subjacent parenchyma, resembling what is observed in light microscopy. Additionally, the epidermal cells were found to be nucleated, contain plastids, numerous mitochondria, and a few oil droplets (Fig. 4a and b).

The subjacent secretory parenchyma cells showed some oil droplets and several plastids containing starch grains. These plastids, in addition to containing large starch grains, are distinguished by their dense stroma and an internal membrane system that forms slightly dilated thylakoids in the peripheral portion (Fig. 4c and d).

#### Early anthesis

**Figure 5:**
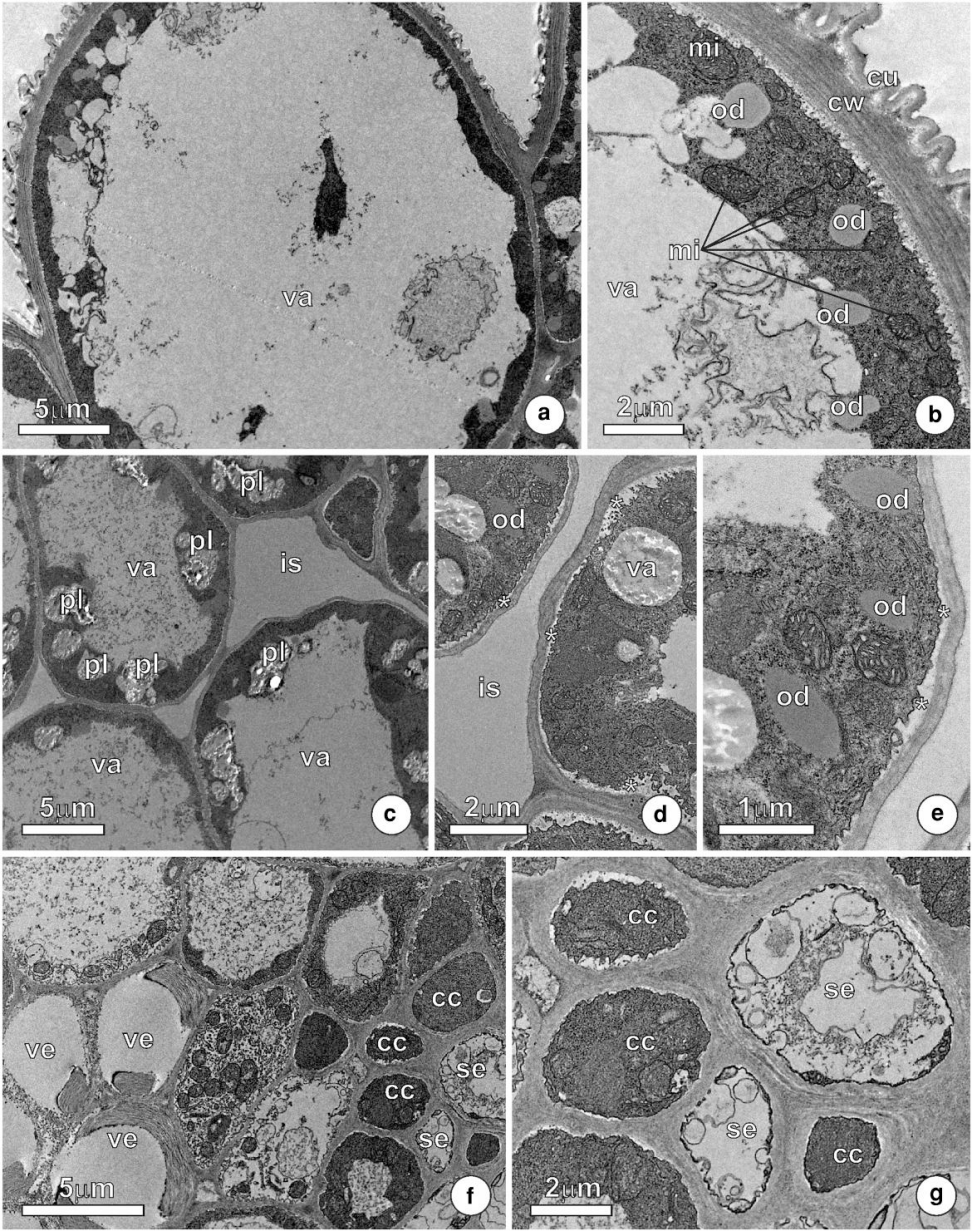
Ultrastructure of the outer filament of *Passiflora pohlii* flower in early anthesis, the stage at which the flower's scent is perceived. a, b) Epidermal cells with a large vacuole and extravascular cytoplasm rich in mitochondria and oil droplets. Note that the cuticle remains attached, with no signs of rupture. c–e) Secretory parenchyma cells. Note, in c), the large central vacuole containing amorphous material; in the plastids, the starch grains are undefined, in an advanced stage of hydrolysis. d) Detail of the peripheral cytoplasm rich in organelles; note the similarity of the vacuolar content with the hydrolysed starch in the previous image. e) Detail of the peripheral cytoplasm with oil droplets and mitochondria; the plasma membrane with undulations and a discrete periplasmic space is created (*). f, g) Detail of the vascular bundle. In f), the xylem (left) presents few vessel elements and parenchyma cells. In the phloem (right), the ordinary companion cells show dense cytoplasm; the sieve tube elements have slightly dense, vesiculated cytoplasm. g) Detail of the phloem with companion cells next to the sieve tube elements with slightly dense and vesiculated cytoplasm. Note the presence of amorphous material with a fibrillar appearance in the sieve tube elements (cc—companion cell; cu—cuticle; cw—cell wall; is—intercellular space; mi—mitochondria; od—oil droplet; pl—plastid; se—sieve tube element; va—vacuole; ve—vessel element).

The epidermal cells maintained a larger central vacuole than the subjacent parenchyma cells. The epidermal cells were characterized by abundant mitochondria, a higher concentration of oil droplets than that observed in preanthesis, and a uniformly distributed smooth endoplasmic reticulum within the dense layer of extravacuolar cytoplasm (Fig. 5a and b).

The subjacent parenchyma cells exhibited plastids and mitochondria as predominant organelles. A marked reduction in the number of starch grains was observed in the plastids; in most of them, the starch was reduced to a shapeless and fibrillar mass (Fig. 5c). There was an increase in the size of the central vacuole, the presence of some plastids devoid of starch, and an increase in the amount of oil droplets compared to the previous stage. The presence of small vacuoles containing amorphous material, similar to that observed in plastids, was striking, with some cases showing a remarkable similarity between plastids and the vacuoles (Fig. 5d). The plasma membrane of many subepidermal cells showed conspicuous sinuosities, suggesting the storage of substances in a periplasmic space (Fig. 5d and e).

In the vascular bundle, a standard structure was observed, with the xylem presenting few vessel elements, characteristically devoid of any organic material (Fig. 5f). In the phloem, the ordinary companion cells had dense cytoplasm; the sieve tube elements had slightly dense and vesiculated cytoplasm, in which the presence of amorphous material with a fibrillar appearance was notable (Fig. 5f and g).

#### Late anthesis

**Figure 6:**
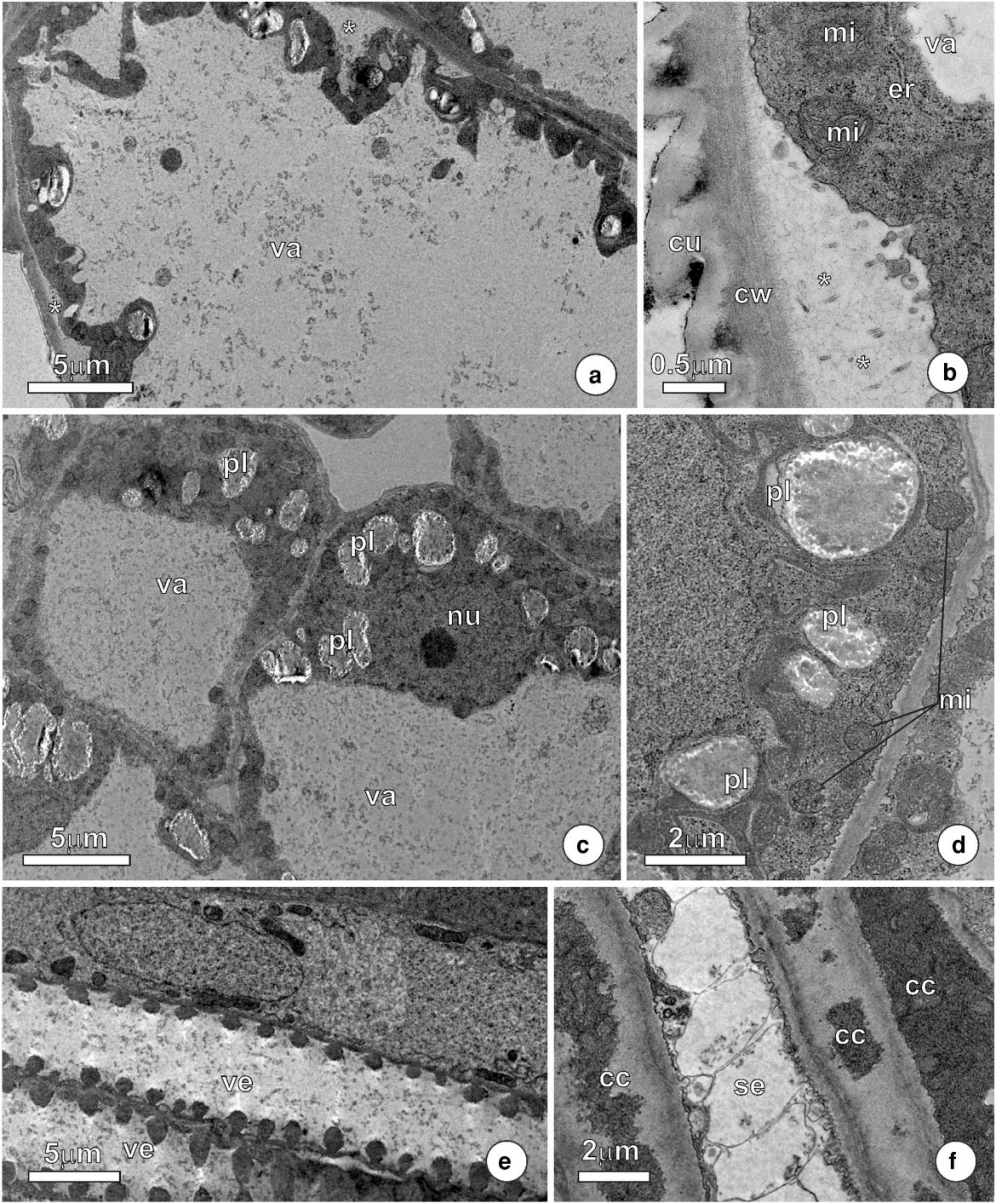
Ultrastructure of the outer filament of *Passiflora pohlii* flower in late anthesis, the stage at which the scent is no longer perceptible. a) Epidermal cell with a large central vacuole and plastids with a few starch granules; the plasma membrane is sinuous, creating a large periplasmic space. b) Detail of the peripheral portion of the epidermal cell, note the intact and well-adhered cuticle; in the periplasmic space (*) there is an accumulation of amorphous material. c) Parenchyma cells with a well-developed nucleus and some amyloplasts with starch remnants. d) Parenchyma cell with some plastids and mitochondria; inside the plastids, there is an amorphous material, residues of already hydrolysed starch. e, f) Detail of the vascular bundle. e) The xylem presents vessel elements that, although already differentiated and dead, are full of substances with an amorphous or fibrillar aspect. f) Detail of phloem, notice the dense cytoplasmic companion cells and a characteristic sieve tube element. (cc—companion cell; cu—cuticle; cw—cell wall; er—endoplasmic reticulum; mi—mitochondria; nu—nucleus, pl—plastid; se—sieve tube element; va—vacuole; ve—vessel element).

In this final stage of anthesis, the epidermal cells lacked the oil droplets present in the previous stages and contained only a small amount of amyloplasts with small starch granules (Fig. 6a). The plasma membrane appeared sinuous, creating a conspicuous periplasmic space, in which amorphous material was observed (Fig. 6a and b). This amorphous material present in the periplasmic space was also observed in the large central vacuole, which at this stage occupies almost the entire protoplast, leaving only a thin layer of extravacuolar cytoplasm (Fig. 6a). Within the subjacent parenchyma, the presence of plastids with remnants of hydrolysed starch is still a striking feature (Fig. 6c and d). Mitochondria and rare segments of endoplasmic reticulum, in addition to the large central vacuole, complete the set of most representative organelles in this phase. The xylem presents vessel elements that, although already differentiated and dead, are filled with substances exhibiting an amorphous or fibrillar aspect (Fig. 6e), whereas in the phloem, the cells retain the same characteristics observed in the previous phase (Fig. 6f).

### Surface structure analysis

**Figure 7:**
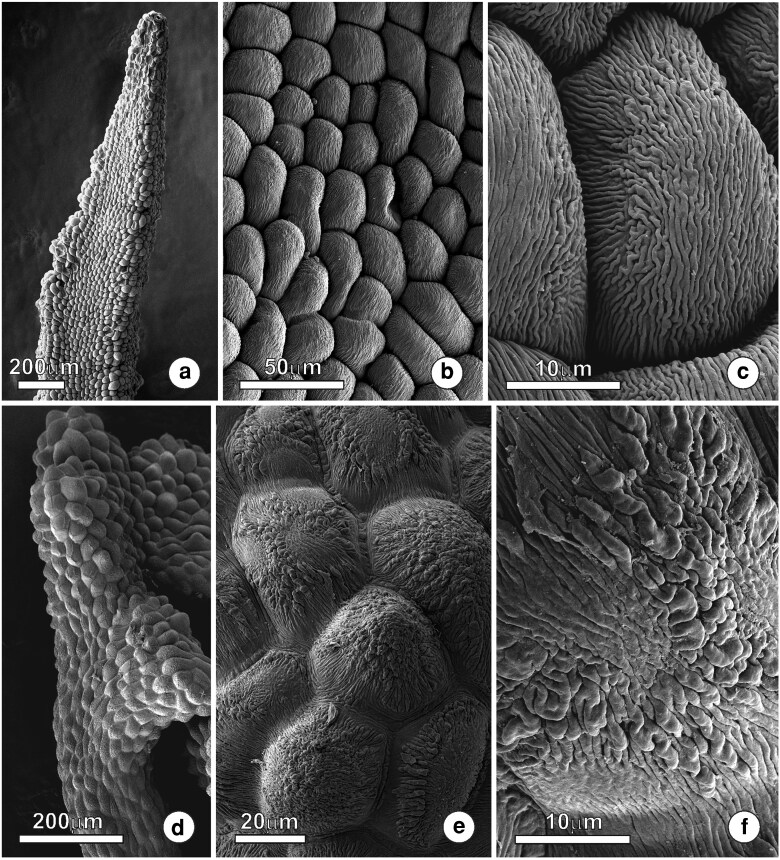
Scanning electron microscopy images of the *Passiflora pohlii* corona filaments in early anthesis. a–c) Outer filament. a) Filament apical zone showing its elliptical tubular form and pointed tips. b, c) The turgid epidermal cells protrude, giving the surface a rough appearance. In c), a detail of the cuticle, with a regular wavy pattern. d–f) Inner filament tip. d) Filament apical zone showing its capitate tips. e) Detail of the epidermal cells, which are less pronounced than those of the outer filament; in f), a cuticle detail, notice the heterogeneous undulation.

Images obtained by scanning microscopy revealed that the epidermal cells of the outer filament of *P. pohlii* were voluminous, projecting onto the surface due to its globoid shape, giving the surface a rough appearance (Fig. 7a and b). Additionally, the cuticle of these cells displayed undulations, significantly increasing the overall surface area (Fig. 7c). Images of the inner filaments revealed capitate tips (Fig. 7d), unlike the pointed tips of the outer filaments. The epidermal cells of the inner filaments (Fig. 7e) were less pronounced compared to those of the outer filaments. However, the cuticle of inner filaments showed undulations, similar to those of outer filaments; the undulations of inner filaments were less homogeneous than those of outer filaments (Fig. 7f).

### Comparative floral volatiles in *Passiflora*

**Figure 8:**
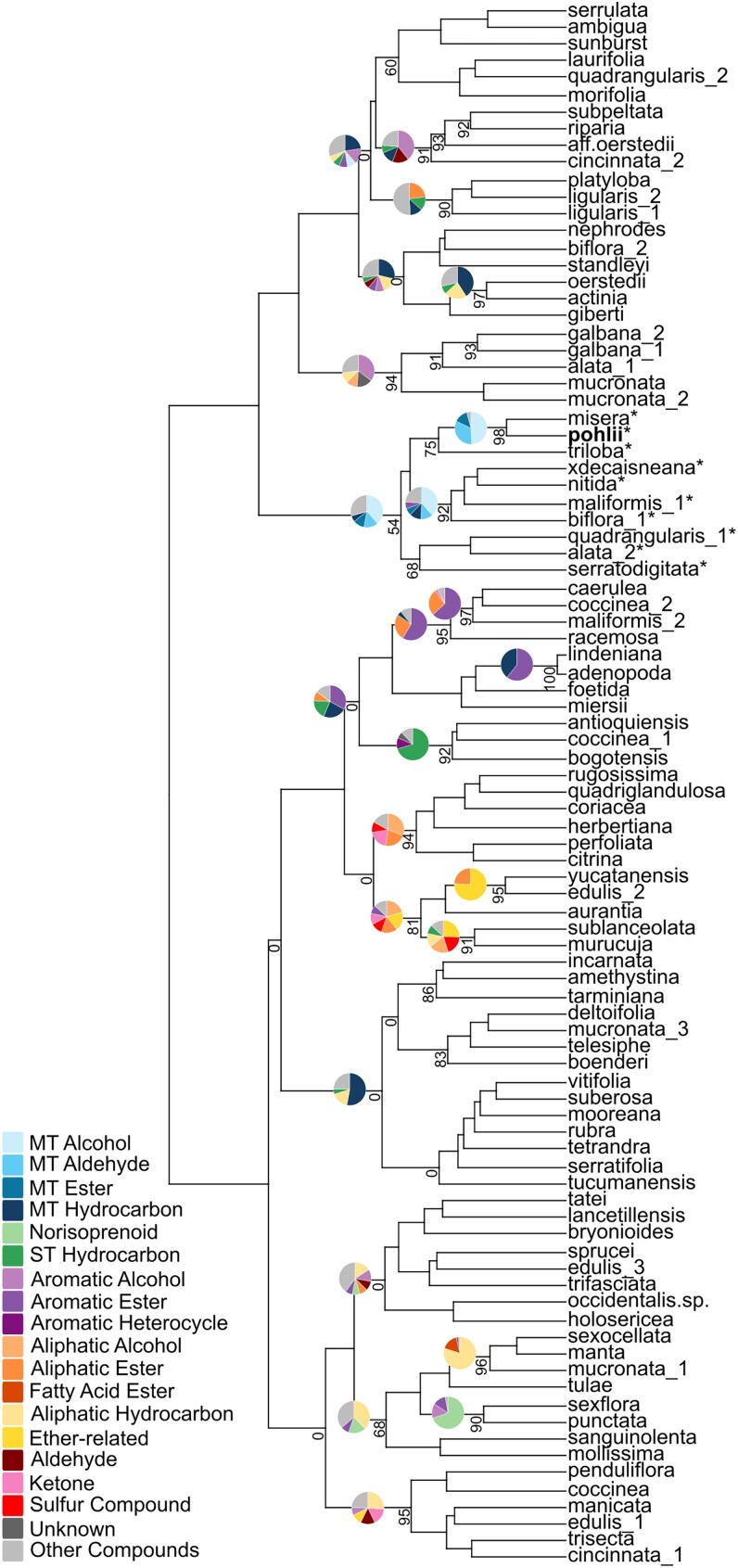
Hierarchical cluster analysis of floral volatile composition in *Passiflora* species. The dendrogram groups species based on similarity in floral scent compound profiles (relative percentages), using literature-derived data and data of the present study. Node circles represent chemical profiles, with size indicating mean relative abundance of dominant compound classes. Branch labels show approximately unbiased (AU) support values (%). Species chemically closest to *Passiflora pohlii* are marked with an asterisk. MT, monoterpene; ST, sesquiterpene.

The analysed 92 datasets of scent profiles from 79 *Passiflora* species summed 318 scent compounds with relative abundance > 2% (see Supporting Information), distributed across 43 functional–structural classes. The compound classes groups with the highest mean relative abundance and highest frequency among species were monoterpenes (MT), aliphatic non-terpenoids, aromatic non-terpenoids, and non-MT terpenoids. Monoterpenes were the most abundant class, with a mean of 36% (±33%) and present in 79% of the species. Among these, monoterpene hydrocarbons reached 26% (±28%; 68% of species) and monoterpene alcohols 14% (±18%; 47% of species). Aliphatic compounds (non-MT) showed a mean of 28% (±27%, 79% of species). Aromatic compounds (non-MT) had a mean of 23% (±25%, 76% of species). Non-MT terpenoids (including sesquiterpenes, homoterpenes, and norisoprenoids) recorded a mean of 15% (±22%, 59% of species). In the dendrogram (Fig. 8), eight branches with robust statistical support (AU ≥ 95%) were identified: one comprising five species, two comprising three species, and the remaining consisted of two species. Among these well-supported branches, the association between *P. pohlii* and *Passiflora misera* had 98% support. Both species exhibited monoterpenes as the main compound class (>90%), including alcoholic monoterpenes, aldehydes, and esters. Although they were similar in terms of compound classes, *P. misera* lacks two of the main compounds of *P. pohlii*: geraniol and geranial.

The clade where *P. pohlii* is inserted (marked with an asterisk, Fig. 8) is the only one where the combination of predominant compound classes consists of alcohol monoterpenes, aldehydes, and esters.

## Discussion

The study demonstrated that the citric floral scent of *P. pohlii* is dominated by terpenoids, especially citronelloid monoterpenes. Quantitative chemical analysis of the floral scent revealed that volatile emission is restricted to the outer filaments of the corona and occurs only for a brief period during the early morning twilight until sunrise, when odour release rapidly declines. The osmophores form a fragrant ring surrounding the centre of the flower, with their secretory cells exhibiting a starch-rich, dense cytoplasm, characteristic of odour-producing tissues.

### Floral scent composition

The floral scent of *P. pohlii* is dominated by acyclic monoterpenes, including citronellol, geraniol, geranial, and neral, collectively termed ‘citronelloids’ (Bergman et al. 2023). These citronelloids originate from geraniol (an alcoholic monoterpene) that undergoes enzymatic transformations to yield other volatile components. For instance, oxidation of geraniol yields geranial (an aldehydic monoterpene), while reduction of one of its double bonds produces citronellol (an alcoholic monoterpene). Subsequent biochemical transformations yield other compounds, such as neral and geranyl acetate (Bergman et al. 2023), which are present in lower proportions within *P. pohlii* scent. This metabolic branching from geraniol is a key differentiating feature of *P. pohlii*, which results in a volatile profile rarely seen in other *Passiflora* species (Fig. 8). However, *P. pohlii* and *P. misera* clustered in the cluster analysis (Fig. 8), reflecting the similarity in the composition of volatile compound types present in their floral scents. Beyond this chemical resemblance, these two species are also phylogenetically close, belonging to the same subgenus (Decaloba) and the same clade (SA8), which exhibits the broadest geographical distribution among Decaloba (Acha et al. 2021). Their floral morphology is also highly similar, characterized by predominantly white flowers, and both are pollinated by bees of the genus *Ptiloglossa*. In the case of *P. misera*, anthesis was described as ‘mainly early and short/dawn-sunset (Amela García and Hoc 2001), which we interpret as being very similar to the anthesis pattern observed in *P. pohlii* in this study. Collectively, this evidence suggests that *P. pohlii* and *P. misera* share not only chemical and morphological characteristics, but also convergent ecological strategies aimed at attracting specific pollinators within a restricted time of day. This pattern appears to extend to other species within Decaloba, as evidenced by *Passiflora biflora*, which shares similar chemical characteristics and colouration, reinforcing the hypothesis of a common evolutionary pattern (Acha et al. 2021).

Besides *P. misera*, the species with chemical profiles similar to those of *P. pohlii* (Fig. 8, red names) exhibit bee-pollinated, diurnal, brightly coloured flowers. On average, these species contain 70% alcoholic-, aldehydic-, and ester-type monoterpenes, compounds that are rare as major constituents in other *Passiflora* species (Fig. 8). In contrast, groups with nocturnal pollinators, such as bats, do not exhibit these compounds as their predominant types; instead, they display aromatic compounds as their main constituents (Fig. 8).

In summary, the data indicate that this distinct scent chemistry is a unique, specialized feature of a small clade (10 species) within *Passiflora*, representing a specific chemical strategy for bee pollination.

### Odour localization

In *P. pohlii*, odour production is a distinctive characteristic, confirmed by both quantitative and ultrastructural analyses to occur exclusively within the outer filaments of the corona (Fig. 6b). This specific localization has also been reported in other *Passiflora* species, through anatomical or sensory methods without chemical confirmation (MacDougal 1994, Amela García 1999, Amela García et al. 2007). However, Varassin et al. (2001) collected odour from the ‘fringe filaments’ (likely the outer filaments) but did not perform ultrastructural analyses of the osmophores or detail odour production studies in relation to the other floral parts. While the rest of the *P. pohlii* flower, including the inner filaments, is virtually scentless, some studies report odour emission from other floral parts, as seen in *Passiflora mooreana*, *P. caerulea* and *Passiflora foetida* (MacDougal 1994, Amela García and Hoc 1998a, 1988b, Amela García 1999). However, these reports are based exclusively on sensory analyses, classifying odour only as mild or intense based on human perception, and lack confirmatory analytical evidence. Despite species-specific variations in reported scent locations, the outer filaments consistently emit scent in all fragrant *Passiflora* species identified.

Positioned at the flower's periphery, the secretory-active tips of the outer filaments facilitate the dispersion of volatiles through wind exposure. Their rough surface and elaborate cuticular ornamentation further enhance volatilization by maximizing surface area. Crucially, these filaments form a continuous osmophore ring encircling the nectary, creating an annular olfactory guide that enables pollinating bees to locate nectar at a fixed centripetal distance, a mechanism convergent with *Antirrhinum majus*, where petal–pollinator contact zones exhibit peak volatile emission (Kolosova et al. 2001). Complementing this olfactory ring, a radial visual nectar guide formed by purple tips on the pleated operculum contrasts with the white filaments, likely serving as an attractive cue for bees (Chittka and Raine 2006) even at low light conditions and potentially directing them towards the central nectary, the primary floral resource sought by *Ptiloglossa* bees in these flowers (Mayorquin A, Schlindwein C, unpublished data).

### Scent emission is restricted at Dawn

Our scent analyses of *P. pohlii* revealed that floral volatiles are primarily emitted during the early phase of anthesis, which occurs in this species at dawn. Shortly after sunrise, the concentration of floral scent decreased by ∼90% (±54%), while the overall volatile composition remained largely unchanged. Despite remaining fully turgid during daylight, the flowers emit little to no scent.

Crepuscular bees of the genus *Ptiloglossa*, whose morning flight activity is limited to the twilight hours (Wcislo and Tierney 2009, Danforth et al. 2019, Liporoni et al. 2020), are the only effective pollinators of *P. pohlii* (Faria and Stehmann 2010). Their visitation schedule coincides with the restricted window of odour production in *P. pohlii*. Floral scent emission synchronized with the pollinator activity period constitutes an adaptive strategy that maximizes the efficiency of volatile compound production, given the high energetic cost of this process. This cost is evidenced by the high accumulation of starch and the increase and prevalence of mitochondria, indicating intense metabolic activity, observed in the osmophores of *P. pohlii*, as well as by the active transport employed to move molecules to the outside of the cell, a mechanism repeatedly demonstrated in angiosperms (Adebesin et al. 2017). Concentrating scent emission within specific temporal windows that coincide with pollinator activity increases the probability of pollination and avoids the waste of chemical signals when pollinators are inactive (Dudareva et al. 2000, Hoballah et al. 2005, Raguso 2008, Brandt et al. 2020). Given the short activity window of *Ptiloglossa* at dawn, we suppose that the precise timing of olfactory signal presentation in *P. pohlii* most likely reflects an evolutionary response driven by these effective crepuscular bee pollinators. Moreover, it may also be related to environmental constraints (Armbruster and McCormick 1990), while also reducing the time for herbivore attraction by floral fragrances (Nunes et al. 2016).

The extended period during which the flowers retain their turgidity in bright sunlight after scent decline at dawn can be viewed as a kind of safety window, allowing them to remain available to eventual diurnal pollinators in case crepuscular bees are absent.

### Structure of the osmophores and secretory process

The *P. pohlii* osmophore exhibits a simple structure, a glandular epithelium overlying a few secretory parenchyma layers. The papillose epidermal cells, a feature consistent with volatile-secreting tissues across other angiosperm species (Sazima et al. 1993, Vogel and Hadacek 2004, Melo et al. 2010, Gonçalves-Souza et al. 2017) and reported in the corona radii of *P. caerulea* and *P. suberosa* (Amela García et al. 2007), support the interpretation of this structure as an osmophore.

The high cytoplasmic density and abundant organelles in the outer filament cortical region are indicative of active secretory function (Tölke et al. 2018, Paiva et al. 2021). This structural feature joins with two key osmophore features, the accumulation of starch in secretory cells (Pansarin et al. 2009, Melo et al. 2010, Tölke et al. 2018) and vacuolar dynamics characterized by volume expansion and flocculent deposits during secretion, a pattern also documented in osmophores of some Anacardiaceae species (Tölke et al. 2018). A similar metabolic strategy occurs in *P. caerulea*, where abundant starch grains at anthesis are rapidly consumed, depleting starch reserves in the post-anthetic stage, concomitant with an increase in cell vacuolation (Amela García et al. 2007). It is important to emphasize that the inner filaments are predominantly composed of highly vacuolated cells and lack detectable metabolic reserves, corroborating the sensory test results and indicating that these structures not have the capacity to synthesize or release volatile compounds.

During late anthesis in *P. pohlii*, small vacuoles containing hydrolysed starch-like material form through plastid-to-vacuole conversion, retaining remnants of degraded starch. These dynamics of organelle interactions and vacuole biogenesis have been documented in nectaries (Peng et al. 2004, Guimarães et al. 2016, Paiva et al. 2021) as well as in osmophores (Gonçalves-Souza et al. 2017) and demonstrate both organellar plasticity and the critical role of starch as an energy source for osmophore metabolism during volatile release, with residual material persisting post-secretion.

Concurrently, the amorphous material observed within vessel elements resembles soluble carbohydrates, suggesting apoplastic translocation of sugars derived from starch hydrolysis or directly from the phloem sieve cells. This observation provides a plausible explanation for the supposed presence of sugar within xylem vessels. Furthermore, the central vascular bundle of the outer filament lacks projections towards secretory cells, and the osmophore secretory tissue itself is nonvascularized, which supports the hypothesis of apoplastic sugar transport. Collectively, this evidence supports the high energy demand of the secretory process commonly associated with osmophores (Gonçalves-Souza et al. 2017). Therefore, it seems reasonable to provide substantial sugar during volatile release, whether for active substance transport, mitochondrial-linked energy production, or biosynthetic processes. Lipid droplets, presumably containing terpenes, were observed from preanthesis onward. Although plastids and endoplasmic reticulum typically mediate terpene synthesis (Langenheim 2003, Rodrigues et al. 2011, Sadala-Castilho et al. 2016), we detected no evidence of synthesis activity during the studied phases. Thus, the cellular structure suggests that the detected terpenes were synthesized earlier, during bud development.

## Conclusion

Odour emission in *P. pohlii* is spatially restricted to a specific floral part (the outer filaments) and temporally optimized to dawn. This process is sustained by starch-derived energy reserves and dominated by geraniol-derived monoterpenes. This strategy, likely combined with visual cues (white colouration), reflects an evolutionary adaptation to pollination by crepuscular bees. However, behavioural bioassays are essential to confirm whether these ‘citronelloids’ function as effective chemical attractants for *Ptiloglossa*. So far, these bees have previously only been shown to respond to benzenoids/phenylpropanoids (e.g. 2-phenylethanol), fatty acid derivatives (1-octanol), and mixtures containing such compounds together with non-‘citronelloid’ terpenes (e.g. linalool) (Martínez-Martínez et al. 2021). The chemical and ecological convergence with *P. misera* and other Decaloba species suggests an evolutionary pattern where ‘citronelloid’ synthesis emerges as a metabolic innovation for efficient pollinator attraction within restricted temporal windows.

## Supplementary Material

plag030_Supplementary_Data

## Data Availability

The raw data are available as supporting information.
